# Solvent‐Mediated Control of the Electrochemical Discharge Products of Non‐Aqueous Sodium–Oxygen Electrochemistry

**DOI:** 10.1002/anie.201601615

**Published:** 2016-05-30

**Authors:** Iain M. Aldous, Laurence J. Hardwick

**Affiliations:** ^1^ Department of ChemistryStephenson Institute for Renewable EnergyUniversity of LiverpoolLiverpoolL69 7ZFUK

**Keywords:** oxygen reduction reaction, peroxides, sodium–oxygen batteries, superoxides, surface-enhanced Raman spectroscopy

## Abstract

The reduction of dioxygen in the presence of sodium cations can be tuned to give either sodium superoxide or sodium peroxide discharge products at the electrode surface. Control of the mechanistic direction of these processes may enhance the ability to tailor the energy density of sodium–oxygen batteries (NaO_2_: 1071 Wh kg^−1^ and Na_2_O_2_: 1505 Wh kg^−1^). Through spectroelectrochemical analysis of a range of non‐aqueous solvents, we describe the dependence of these processes on the electrolyte solvent and subsequent interactions formed between Na^+^ and O_2_^−^. The solvents ability to form and remove [Na^+^‐O_2_^−^]_ads_ based on Gutmann donor number influences the final discharge product and mechanism of the cell. Utilizing surface‐enhanced Raman spectroscopy and electrochemical techniques, we demonstrate an analysis of the response of Na‐O_2_ cell chemistry with sulfoxide, amide, ether, and nitrile electrolyte solvents.

Intensive research into lithium–oxygen (Li‐O_2_) batteries in recent years has led to the study of alternative alkali‐metal–oxygen cell chemistries.^1^ The inclusion of other alkali metals in the research of energy storage devices beyond lithium ion batteries is merited by enhancing sustainability, yet still providing striking theoretical values for specific energy (Na‐O_2_ 1505 Wh kg^−1^ K‐O_2_ 1100 Wh kg^−1^). The possible reduced oxygen discharge products for M‐O_2_ electrochemistry include superoxide (MO_2_), peroxide (M_2_O_2_), and oxide (M_2_O; M=alkali metal) species.^2^ With the exception of one recent study detecting LiO_2_,^3^ the main discharge product of Li‐O_2_ is Li_2_O_2_.^4^ However for Na‐O_2_ batteries, both Na_2_O_2_⋅2 H_2_O^5^ and NaO_2_ 1b, 6 have been reported, along with KO_2_ 1a for K‐O_2_ batteries.

Although well documented for Li‐O_2_,^7^ Na‐O_2_ electrolytes have thus far been limited to carbonate, ether, and the ionic liquid (IL) *N*‐methyl‐*N*‐propylpiperidinium bis(trifluorosulfonyl) imide (PP_13_TFSI)‐based electrolytes.6a,6d Carbonate and the IL have been shown to be unstable electrolytes for NaO_2_.^8^ Carbonate‐based electrolytes were initially shown to give Na_2_O_2_ as the discharge product, however sodium carbonate and carboxylates have now been detected more recently, and thought to be the major discharge product.^8^ These observations match the behavior observed in Li‐O_2_ cells cycled in organic carbonates as the main solvent.6d Glyme‐based electrolytes; including tetraethylene glycol dimethyl ether (TEGDME) and diethylene glycol dimethyl ether (DEGDME) have shown evidence of NaO_2_, Na_2_O_2_, and Na_2_O_2_⋅H_2_O products.6a, 8 One major aspect of metal–O_2_ battery enquiries is the inducement of a solution‐based mechanism to enhance discharge capacity.6a, 9 This is achieved through the solvation of superoxide or control of the Lewis acidity of the alkali metal cation, and thus the strength of alkali‐metal–superoxide interactions.9b, 10

Solvation of superoxide through controlled water content increases discharge product size, including toroidal Li_2_O_2_ 9a and cubic NaO_2_.6a, 11 Further understanding of this process in Na‐O_2_ has established that the electrolyte acidic proton content promotes the formation of HO_2_ radicals as a phase transfer catalyst.6a, 12 According to Xia et al.,6a HO_2_ enables the removal of superoxide from the surface above 5 ppm H_2_O/H^+^ content and a subsequent solution metathesis reaction with Na^+^ creates NaO_2_ nuclei that precipitate on the electrode surface. Similarly, a recent study by Jirkovsky et al.^13^ stated that even small amounts of water (10–16 ppm) enhances the oxygen reduction reaction (ORR) kinetics. The suggested mechanism defined the role of water as an active part of surface intermediates, through hydrogen bonding to LiO_2_, which promotes the formation of Li_2_O_2_ and the resulting partial dissociation of H_2_O to HO_2_ and OH^−^.^13^ Superior discharge capacity has been demonstrated with benzoic and acetic acid in Na‐O_2_, along with phenol and ethanol in Li‐O_2_, providing additional evidence for this phenomenon.6a,6b

In very dry electrolytes (≤10 ppm), control of the Lewis acidity of the alkali metal cation through electrolyte solvation allows alkali‐metal–superoxide ion pairs to form and react within the double layer to enhance discharge capacity.9b Once in solution, LiO_2_ may undergo a second electron addition or a chemical disproportionation reaction to form Li_2_O_2_. These observations were qualitatively compared to Guttmann donor number, whereby high donor number solvents, including dimethylsulfoxide (DMSO), are able to better support a solution‐based mechanism enhancing battery capacity.9b Mid‐ and low‐ranged donor number solvents lack the solvation power to support a solution‐based mechanism.9b By utilizing in situ surface‐enhanced Raman spectroscopy (SERS) as an interfacial probe, we investigated the effect of solvent donor number upon the oxygen reduction reaction (ORR) in the presence of sodium cations.

A detailed electrochemical study is presented within the Supporting Information (Figures S1 and S2, Tables S1–S4). Variations in the CV response were observed that were dependent both on the electrode substrate and the solvent; however, limited mechanistic insights could be directly acquired. SERS data provides insight into surface species and intermediates. By applying this technique to each electrolyte system, it is apparent that solvent choice can strongly affect the identity of Na_*x*_O_*y*_ species on planar roughened Au electrodes. Upon discharge, the high donor number solvents, DMSO and DMA, produced signals in the region for O_2_^−^ and NaO_2_ (Figure 1 a,b and Table 1). This agrees with the electrochemical analysis for DMSO, in which only a small variation in the CV response is noted for the exchange of TEA^+^ with Na^+^. The corresponding Raman spectra for systems in the absence of alkali metal cations only displayed a signal at 1110 cm^−1^ for the O−O stretch of O_2_^−^ adsorbed on the surface.9b There was little change in the spectra upon discharge after moving to DMA. The same formation of O_2_^−^ and subsequent NaO_2_ formation was observed. A blue shift of approximately 5–10 cm^−1^ was identified from the expected values of 1110 cm^−1^ (υ_O−O_, O_2_^−^) and 1156 cm^−1^ (υ_O−O_, NaO_2_), denoting varying interactions of O_2_^−^ and cation between different solvents.9b, 14

**Figure 1:**
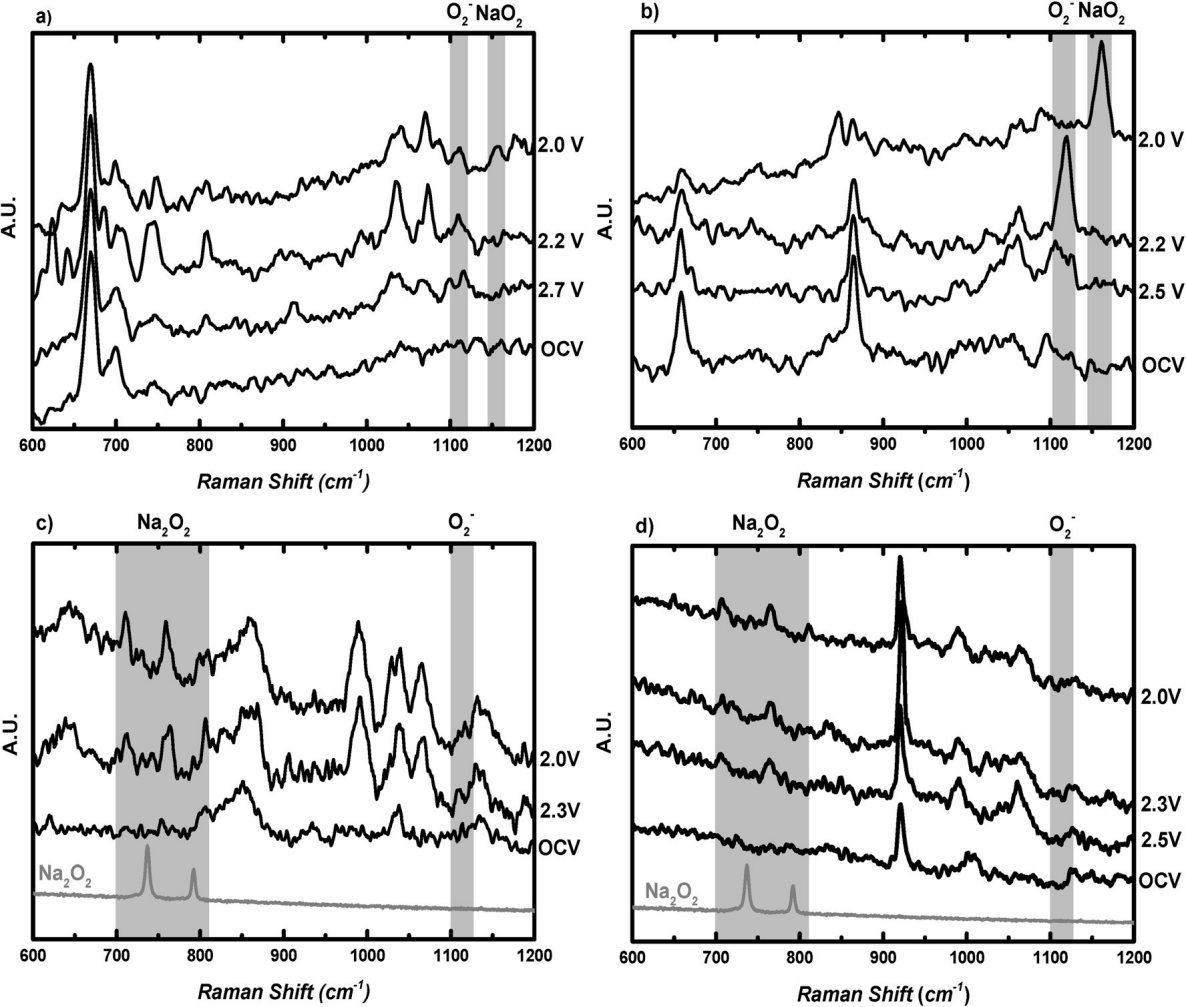
In situ SERS of oxygen‐saturated 0.1 m NaOTf in a) DMSO, b) DMA, c) 1 m NaOTf in DEGDME, and d) 0.1 m NaOTf in MeCN and roughened Au working disc electrodes at 23 °C, 0.1 V s^−1^ at varying potentials vs. Na^+^/Na.

**Table 1:** Raman bands for ORR discharge products/cm^−1^.

	O_2_^−^ (Au‐O_2_)	Na_2_O_2_ (736 cm^−1^)	Na_2_O_2_ (792 cm^−1^)	O_2_^−^ (O_2_^−^)	NaO_2_ (1156 cm^−1^)
DMSO	488	–	–	1107	1156
DMA	–	–	–	1119	1161
DEGDME	–	710	760	1109	–
MeCN	–	706	764	1108	–

Within these systems, superoxide initially forms in the presence of Na^+^, as shown in Equations (1) and (2).

(1) $$O{}_{2(\mathrm{ads})}+e{}^{-}⇌O{}_{2}{}^{-}{}_{\left(\mathrm{ads}\right)}$$

(2) $$\mathrm{Na}{}^{+}+O{}_{2}{}^{-}{}_{\left(\mathrm{ads}\right)}⇌\left[\mathrm{Na}{}^{+}\text{-}O{}_{2}{}^{-}\right]{}_{\left(\mathrm{ads}\right)}$$

(3) $$\left[\mathrm{Na}{}^{+}\text{-}O{}_{2}{}^{-}\right]{}_{\left(\mathrm{ads}\right)}\to \left[\mathrm{Na}{}^{+}\text{-}O{}_{2}{}^{-}\right]{}_{\left(\mathrm{sol}\right)}$$

The addition of an electron to the surface‐adsorbed O_2_ leads to the formation of superoxide that subsequently interacts with Na^+^ at the interface. This interaction is heavily dependent on the solvation of both O_2_^−^ at the surface and the solvation of Na^+^. These interactions control the acidity and basicity of the Na^+^ and O_2_^−^. The solvation shell in these cases will consist of anions (O_2_^−^ and OTf^−^), cation (Na^+^), and solvent molecules.^15^ The OTf^−^ presence is based on the solubility of the salt within each solvent, which induces the formation of contact, solvent‐separated, and free ion pairs in solution.^16^ In DMSO, the peak at 1032 cm^−1^ (Table S5) splits at a potential of 2.0 V versus Na^+^/Na denoting ion pair formation, but does not observably affect the Na_*x*_O_*y*_ peaks within the spectra.

The interaction between DMSO‐solvated [Na^+^‐O_2_^−^] is highly favorable, allowing the interaction to be ascribed to an ion pair, which corresponds to the detection of the band at 1107 cm^−1^. This soluble species is easily removed from the surface, which explains the quasi‐reversible nature of O_2_ and Na^+^ electrochemistry in DMSO. Furthermore, the detection of the signal for NaO_2_ at 1156 cm^−1^ is likely due to the aggregation and precipitation of NaO_2_ on the surface as the reductive potential increases to 2.0 V versus Na^+^/Na. Multiple CV scans within 0.1 m NaOTf showed that the quasi‐reversible process breaks down, and revealed the formation of two oxidation peaks within the initial cyclovoltammetric peak (Figure S3). This corroborated the initial ion pair formation, and the subsequent aggregation and precipitation of NaO_2_ on the surface. As the number of CV sweeps increases, more time has been allowed for NaO_2_ to precipitate, which leads to the growth of the second oxidation peak at 2.75 V versus Na^+^/Na.

A similar situation is induced by DMA‐solvated NaOTf, but the change in donation from solvent to cation the Lewis acidity of sodium that may explain the distinct shift from [Na^+^‐O_2_^−^] to NaO_2_ (1119 cm^−1^ to 1161 cm^−1^) within the spectra. The mechanism here is considered to be as stated in Equations (1) and (2). This follows the removal of NaO_2_ from the surface, subsequent electrolytic saturation within the double layer, and aggregation and precipitation of NaO_2_ (Scheme 1). However, considering the water content of these systems (≤20 ppm) and recent data by Xia et al.,6a the proposed inducement of this reaction through HO_2_ formation should enhance the formation of cuboid NaO_2_. Therefore, solvents can induce the removal of Na_*x*_O_*y*_ from the surface in a similar manner to how water can solvate and remove O_2_^−^ from the surface, as suggested by Xia et al.6a However, we have found no spectroscopic evidence, either indirect or directly, of the presence of H_2_O or HO_2_, as discussed in detail by both Xia et al.6a and Jirkovsky et al.^13^

**Scheme 1:**
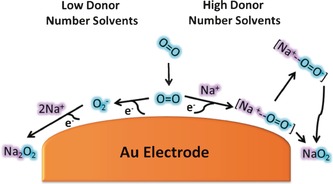
Mechanism of oxygen reduction in non‐aqueous solvents in the presence of Na^+^ cations (with H_2_O ≤20 ppm).

The poor conductivity of low concentration salt (0.1 m), DEGDME‐based electrolyte required that the Raman analysis be carried out at 1 m NaOTf in order to reduce significant overpotentials (Figure 1 c). The SERS data showed that the main discharge product was Na_2_O_2_, based on the observation of a shifted doublet peak at 710 and 760 cm^−1^. This is in contrast to the majority of Na‐O_2_ cell observations on a number of bulk carbon electrodes in which NaO_2_ is the major product identified.6c, 11, 14, 17 The detection of Na_2_O_2_ rather than NaO_2_ on a planar roughened gold electrode may be due to surface morphology as the current role of the surface is unclear in directing preferential formation of NaO_2_ or Na_2_O_2_.17a DEGDME is a medium donor number solvent that in Li‐O_2_ chemistry has been shown to support a solution‐based mechanism and surface mechanism by increasing the longevity of LiO_2_. Here, it is believed that a different process is occurring. In this case, the lifetime and energetics of the ion pair of [Na^+^‐O_2_^−^] may increase the amount of Na_2_O_2_ formed owing to the transfer of a second electron, but also owing to the kinetic and thermodynamic stability of the products.17a Na_2_O_2_ has been shown to be the thermodynamically favorable product over NaO_2_ above 10 μm.17a

MeCN‐based electrolyte SERS data also displayed a doublet band at 714 and 767 cm^−1^ that was assigned as Na_2_O_2_ (Figure 1 d). The absence of a NaO_2_ signal indicated preferential Na_2_O_2_ formation, suggesting that any initially formed NaO_2_ is short‐lived or that superoxide is solely present before a second electron transfer. Therefore, if a surface‐bound NaO_2_ film is present, then it rapidly grows beyond kinetic stability, allowing for a second electron reduction and a subsequent Na_2_O_2_ discharge product.17a The increased Lewis acidity of Na^+^ in MeCN causes the formation of a dense passivation film of Na_2_O_2_, which is comparable to the behavior observed in Li^+^ in the same solvent (Figure S4). The assigned Na_2_O_2_ signals here were shifted from our Raman standard. This is a similar case for DEGDME (Figure 1 c), which we will explain further below.

To confirm that the shifted bands assigned as Na_2_O_2_ were due to reduced oxygen species, careful control SERS experiments were carried out. Electrolytes purged under Ar did not show any signals assigned to reduced O_2_^−^ species as above (Figure S5–S8). In the same spectral region as the higher Na_2_O_2_ peak in the SERS data, there was a corresponding OTf^−^ anion peak at 760 cm^−1^ at OCV, however there are no peaks that appear around 710 cm^−1^. The OTf^−^ band does not change in intensity under potential control, and so therefore this feature arises from Na_2_O_2_ due to O_2_ species. The presence of interfacial OTf^−^ can be explained by the formation of ion pairs or aggregation of ion pairs at the interface. The doublet feature assigned to Na_2_O_2_ is shifted to a lower wavenumber than expected. If this shift is due to H_2_O, then it would be expected to increase the signal to a higher wavenumber.^18^ The strong presence of ion‐pairs within the double layer may enhance the interaction of surface Na_2_O_2_ and the OTf^−^ anion, which could of explain the observation of the red‐shifted bands (by ca. 30 cm^−1^) of Na_2_O_2_.

The formation of ion pairs was revealed by the appearance of a peak at 1040 cm^−1^. This region denotes the ν_as_ SO_3_, which is considered the group within the anion that interacts with alkali metal cations.^16^, ^19^ The formation of ion‐pairs interacting with Na^+^ within Na_2_O_2_ at the surface may cause this observed shift. These bands relating to ion‐pair formation are detected in DMSO and DEGDME, suggesting that ion solvation may influence the discharge product.

Therefore, the SERS data provides spectroscopic evidence that the lack of solvation of O_2_^−^ in low donor number solvents increases the proximity of O_2_^−^, allowing for a second electron reduction to form a thin passivating film of Na_2_O_2_ [Eq. (4), (5)] (Scheme 1). This mechanism follows:

(4) $$O{}_{2(\mathrm{ads})}+e{}^{-}⇌O{}_{2}{}^{-}{}_{\left(\mathrm{ads}\right)}$$

(5) $$2\;\mathrm{Na}{}^{+}+e{}^{-}+O{}_{2}{}^{-}{}_{\left(\mathrm{ads}\right)}\to \mathrm{Na}{}_{2}O{}_{2}$$

In conclusion, in situ SERS investigations have shown that solvent choice can influence the overall surface discharge product of Na‐O_2_ cell chemistry. Observable, yet shifted, SERS signals for Na_2_O_2_ in low donor number solvents suggest that solvation of initially formed O_2_^−^ is important in the control of this mechanism on Au electrodes. Higher solvation leads to the absence of Na_2_O_2_ owing to initial formation of an ion pair between Na^+^ and O_2_^−^_(ads)_, which is removed from the surface and then aggregates and precipitates out later as NaO_2_ in the discharge process. Solvents with a lower ability to control the Lewis acidity of Na^+^ do not form an ion pair interaction with O_2_^−^ and proceeds through a surface mechanism where, upon further oxygen reduction, Na_2_O_2_ is preferentially formed at the interface.

## Supporting information

As a service to our authors and readers, this journal provides supporting information supplied by the authors. Such materials are peer reviewed and may be re‐organized for online delivery, but are not copy‐edited or typeset. Technical support issues arising from supporting information (other than missing files) should be addressed to the authors.

Supplementary
