# Supplementary material for: Solvent‐Mediated Control of the Electrochemical Discharge Products of Non‐Aqueous Sodium–Oxygen Electrochemistry
Source: Angew Chem Int Ed Engl. 2016 May 30;55(29):8254–7. doi: 10.1002/anie.201601615 (PMC4999043; doi:10.1002/anie.201601615)
Supplement: Supplementary file 1 — Supplementary [file ANIE-55-8254-s001.pdf]

Angewandte Chemie
Eine Zeitschrift der Gesellschaft Deutscher Chemiker

Supporting Information

Solvent-Mediated Control of the Electrochemical Discharge Products of Non-Aqueous Sodium–Oxygen Electrochemistry

*Iain M. Aldous and Laurence J. Hardwick**

anie_201601615_sm_miscellaneous_information.pdf

Supporting Information

Contents

Methods Section

Voltammetric Analysis

Tables

Table S1 –Cyclic voltammetric data from Au working disc electrodes in oxygen enriched 0.1 M TEA OTf in various solvents at 23 °C, 0.1 Vs^{-1}

Table S2 –Cyclic voltammetric data from Au working disc electrodes in oxygen enriched 0.1 M Na OTf in various solvents at 23 °C, 0.1 Vs^{-1}

Table S3 –Cyclic voltammetric data from GC working disc electrodes in oxygen enriched 0.1 M TEA OTf in various solvents at 23 °C, 0.1 Vs^{-1}

Table S4 –Cyclic voltammetric data from GC working disc electrodes in oxygen enriched 0.1 M Na OTf in various solvents at 23 °C, 0.1 Vs^{-1}

Table S5 - Corresponding Solvent Raman Bands

Table S6 - Donor number and ^{23}Na NMR peak shift for correlated data in Figure S3

Figures

Figure S1 - Voltammetric study of OER/ORR in oxygen enriched 0.1M TEA OTf & 0.1M NaOTf using DMSO, DMA, DEGDME, MeCN and PC on GC, 100 mV s^{-1}, 23°C

Figure S2 - 20 voltammetric cycles study of OER/ORR in oxygen enriched 0.1M NaOTf using DMSO, Au, 100 mV s^{-1}, 23°C

Figure S3 Voltammetric cycling of of OER/ORR in oxygen enriched 0.1M Na OTf DMSO on Au, 100 mV s^{-1}, 23°C

Figure S4 Voltammetric comparison of OER/ORR in oxygen enriched 0.1M NaOTf and LiOTf in MeCN on Au, 100 mV s^{-1}, 23°C

Figure S5. *In situ* SERS of argon enriched 0.1 M NaOTf in DMSO

Figure S6. *In situ* SERS of argon enriched 0.1 M NaOTf in DMA

Figure S7 *In situ* SERS of argon enriched 0.1 M NaOTf in DEGDME

Figure S8 *In situ* SERS of argon enriched 0.1 M NaOTf in MeCN

Figure S9 Infrared spectrum of NaOTf Powder (Aldrich)

Figure S10 Oxidation reduction cycle procedure showing increasing intensity of pyridine at a Au interface

Figure S11 ^{23}Na NMR correlated to Gutmann donor number for various non-aqueous solvents and water in 0.2 M $NaClO_4$ internally referenced against 2 M NaCl

References

Methods Section

Solvent purification: All solvents were purified by distillation over CaH_2. The distillate of which was then dried over freshly activated molecular sieves (4 Å) reducing the water content to a value of ≤ 5ppm water. This was determined using a coulometric Karl Fischer titrator (Mettler-Toledo). Tetraethylammonium trifluoromethanesulfonate (TEAOTf) (≥ 99.0 %, Aldrich) and sodium trifluoromethanesulfonate (NaOTf) were dried under vacuum at 120 ^oC for 16 hours before use. It has previously been noted that the purity of commercial NaOTf contains impurities such as NaOH and hydrates. However, characterisation by FTIR (**Fig S9**) of the commercial powder found no such impurity and therefore the salt was without further purification.

Electrochemical cells were prepared by washing and sonicating in acetone (ACN) and Milli-Q water (18.2 M **Ω**) and dried at 120^oC under vacuum for 12 hours. Electrochemical measurements were carried out within a glass, multi necked air tight cell within an inert atmosphere Glovebox providing less than 50 ppm O_2 and 0.1 ppm H_2O at ambient temperature. High purity oxygen (≥99.999%) and argon (≥99.998%) further dried with a water trap and desiccant drying tube was used to degas and oxygenate electrolytes via gas inlet and outlets. Polycrystalline gold (Au), glassy carbon (GC) working disc electrodes (0.3 cm diameter) were used as working electrodes. The electrode surfaces were polished mechanically with decreasing grain sized alumina slurries (1.0 µm, 0.3 µm and 0.05 µm). These were washed in Milli-Q water (18.2 M **Ω**) and sonicated between slurries and before being dried at 120 ^oC under vacuum overnight. Before the electrochemical cells were setup, all electrodes were rinsed with dried electrolyte containing ≤ 5 ppm water. A platinum coiled wire was used as a counter electrode and a silver wire as a quasi-reference electrode. The quasi-reference electrode was standardised against an internal ferrocene reference which has a potential of + 0.4 V vs. NHE. These were prepared in a similar manner to the electrochemical cell accepting the counter electrode that was additionally flame annealed before use electrochemical techniques were carried out at 25 ^oC.

In situ SERS measurements were setup similarly with a glass, multi-necked gas-tight cell fitted with a sapphire window. A millimetre behind this window an electrochemically roughened Au working electrode was placed. The Au working electrode was roughened using an oxidation/reduction cycle (ORC) described previously[1] (**Fig S10**) shows the effect of applying the ORC (oxidation-reduction cycle) treatment to a Au electrode with pyridine drop cast on the electrode. Beyond 25 cycles a reduction in signal intensity is observed. Spectra were recorded using a 50x objective on a Raman spectrometer (Renishaw In via) with a 633 nm laser (2 mW) calibrated against a silicon wafer.

^{23}Na NMR studies were carried out within an in house sealed NMR tube on a Bruker Ultrashield 400 MHz spectrometer. The setup contained a 2M NaCl standard solution in a sealed capillary tube which was dried overnight before use and put in the glovebox a week before using. Along with this 0.2M $NaClO_4$ in the corresponding solvent was added to the NMR tube for testing. Both the spectrum from ^{23}Na and ^{1}H NMR was taken as a control measure and the shift standardised to that of the NaCl peak within the spectra.

Fourier Transformed Infrared (FTIR) spectroscopy was carried out upon a Nicolet iS50 FT-IR. The transmission spectrum was taken using a pelletised mix of caesium bromide and the compound in question.

Voltammetric Analysis

The electrochemistry of oxygen was investigated using cyclic voltammetry upon polycrystalline Au and GC within sodium triflate (Na OTf) dissolved with in four standard electrolyte solvents: dimethylsulfoxide (DMSO) dimethylacetamide (DMA), diethylene glycol dimethyl ether (DEGDME) and acetonitrile (MeCN).

Voltammetric peak to peak separation offers little in terms of reversibility trend between electrolytes (Table S1), however there is a clear visual decrease in the ratio of charge and peak current as you switch the non-aqueous solvent with the trend of reversibility DMSO > DMA > DEGDME > MeCN (see

Tables S1 and S2 on Au working electrode and **Tables S3 and S4** using GC working electrode). As a comparative electrolyte property Gutmann donor number (G.D.N. **Fig**. **S11**) is useful to ascertain the differences between the chemistries of these electrolytes. Although this is an important consideration other factors may be pivotal in the underlying fundamentals of this mechanism. This may include the solubility and diffusion of NaO_2 in these solvents as well as the viscosity of solvent.[2] High G.D.N. solvents in TEA^+ and Na^+ electrolytes, DMSO and DMA G.D.N. of 29 and 26 as calculated by ^{23}Na NMR (**S3**)) show relatively little change upon the reduction of oxygen. The similar quasi-reversibility is maintained if only with a small change in peak position, a 12 mV increase in hysteresis from TEA OTF to Na OTf, and a decrease of 1% the ratio of Q_a/Q_c as shown in table S3 and S4 for DMSO. Our study extends itself to GC working electrodes and establishes similar voltammetric features.

The observable difference between the two surfaces is a lesser change in the Q_a/Q_c ratio of the process from 75% to 71%. Another difference is the formation of a secondary peak in the reduction response on Au what is broader if not present on GC surfaces. This is similar to the Li-O_2 voltammetric response, whereby there is the formation of LiO_2 in the initial voltammetric peak and subsequent formation of Li_2O_2 in secondary peak. Here due to the absence of any signals for Na_2O_2 detailed in out SERS analysis this secondary peak is feasibly caused the formation of NaO_2 precipitated on the surface. The same process is occurring to a lesser extent on the GC electrode, but with a lesser tendency for adsorption on the surface of the discharge products of this reaction this feature is broad and diminished.

The surface dependent kinetics of dioxygen redox chemistry perhaps hinders the reaction, at this scan rate, affecting the charge ratio of the peak/peak current ratio more greatly on Au electrode surfaces. The greater propensity for surface adsorption of reactants and electrolyte components at this interface is a secondary influence on the calculated parameters.

The proposed explanation is that the Lewis acidity of DMSO solvated NaO_2 induces an ion pair interaction between Na^+ and O_2^- resembling the quasi reversible electrochemical response of [TEA^+--O_2^-] ion pairs within the electrolytes (0.1 M Na OTf: 0.18 V TEA OTf: 0.17 V peak separations). DMA

based electrolytes with Na^+ observe a decreased current density on discharge and a positively shifted doublet upon charge (2.64 V & and 2.81 V). The reduction peak decreasing in current density by a factor of 2 from -2.7 mA cm^{-2} to -1.38 mA cm^{-2}. The unchanging shift of which concludes that the process, at least on reduction, is most likely a similar [Na^+--O_2^-] interaction as in DMSO. Due to the slight decrease in Lewis acidity, and subsequent decrease in the favourability of the interaction between the Na^+ and O_2^-, it suggests a lesser solubility of NaO_2 and therefore enhanced tendency for precipitation on the surface. This explains the decreased current density but non-shift of the oxidation peak current. The supporting SERS conclusions from this data suggest a lone discharge product of NaO_2, which is not supported by the electrochemistry's complex charging characteristics, with peaks arising at 2.64 V and 2.81 V. More investigation is needed to ascertain the mechanism on charge however at present it is presumed these features are due to NaO_2 and [Na^+--O_2^-] species..

Upon discharge as solvent donor number decreases there is a further decrease in peak current density observed. For MeCN this is a decrease of 84% which shows almost total passivation of the electrode surface upon reduction. Similar characteristics for DEGDME are seen when comparing Na OTf electrolytes with TEA OTf electrolytes. However, the reduction in peak current density is only 36% on Au surface displaying the gradual increase in surface passivation upon decreasing solubility or solvent stabilisation of NaO_2. However this ratio is masking a reduced peak current for oxygen reduction in TEA OTf electrolytes. The passivation is occurring within this electrolyte may be of similar behaviour to that discussed by Johnson *et al*[3] in there study of Li-O_2 battery electrolytes. Here they discuss competing surface and solution based mechanisms within DEGDME through the limited ability of the solvent to stabilise and solvate LiO_2 removal from the surface. This subsequently increases discharge capacity before passivation occurs through surface formed and solvent deposited Li_2O_2.

If applied to this study with sodium electrolytes interestingly the surface passivation of GC is much more enhanced in this electrolyte observing 72% reduction in peak current comparing Na OTf and TEA OTf electrolytes within this media. Charging characteristics observe little response compared to high

donor number solvents, but a similar positive peak shift in oxidative processes with a number of peaks observed. It is speculated these peaks may arise from oxidation of NaO_2 and Na_2O_2, but further experiments are needed to confirm the identity of these species.

Table S1: Cyclic Voltammetric Data from Au Working Disc Electrodes in Oxygen enriched 0.1 M TEA OTf in various solvents at 23 °C, 0.1 Vs^{-1}

	I_{pc} /mA cm^{-2}	I_{pa} /mA cm^{-2}	Ep_c Vs. [Na/Na^+]/V	Ep_a Vs. [Na/Na^+]/V	ΔEp Vs. [Na/Na^+]/V	Charge Ratio (Q_a/Q_c)	Peak Current Ratio (I_{pa}/I_{pc})
DMSO	-0.49	0.35	2.41	2.73	0.32	0.71	0.71
DMA	-2.7	2.27	2.13	2.32	0.19	0.79	0.84
DEGDME	-0.88	0.51	2.16	2.58	0.42	0.72	0.58
MeCN	-3.37	1.93	1.66	2.53	0.9	0.65	0.57

Table S2: Cyclic Voltammetric Data from Au Working Disc Electrodes in Oxygen enriched 0.1 M Na OTf in various solvents at 23 °C, 0.1 Vs^{-1}

	I_{pc} /mA cm^{-2}	I_{pa} /mA cm^{-2}	Ep_c Vs. [Na/Na^+]/V	Ep_a Vs. [Na/Na^+]/V	ΔEp Vs. [Na/Na^+]/V	Charge Ratio (Q_a/Q_c)	Peak Current Ratio (I_{pa}/I_{pc})
DMSO	-0.56	0.3	2.22	2.66	0.44	0.70	0.53
DMA	-1.38	-	2.147	-	-	-	-
DEGDME	-0.57	-	1.97	-	-	-	-
MeCN	-0.48	-	2.32	-	-	-	-

Table S3: Cyclic Voltammetric Data from GC Working Disc Electrodes in Oxygen enriched 0.1 M TEA OTf in various solvents at 23 °C, 0.1 Vs^{-1}

	I_{pc} /mA cm^{-2}	I_{pa} /mA cm^{-2}	Ep_c Vs. $[Na/Na^+]$/V	Ep_a Vs. $[Na/Na^+]$/V	ΔEp Vs. $[Na/Na^+]$/V	Charge Ratio (Q_a/Q_c)	Peak Current Ratio (I_{pa}/I_{pc})
DMSO	-0.63	0.48	2.4	2.56	0.16	0.68	0.76
DMA	-2.71	2.27	2.13	2.32	0.19	0.97	0.83
DEGDME	-1.68	1.22	1.42	2.39	0.97	0.70	0.72
MeCN	-4.98	3.2	2.14	2.3	0.16	0.88	0.64

Table S4: Cyclic Voltammetric Data from GC Working Disc Electrodes in Oxygen enriched 0.1 M TEA OTf in various solvents at 23 °C, 0.1 Vs^{-1}

	I_{pc} /mA cm^{-2}	I_{pa} /mA cm^{-2}	Ep_c Vs. $[Na/Na^+]$/V	Ep_a Vs. $[Na/Na^+]$/V	ΔEp Vs. $[Na/Na^+]$/V	Charge Ratio (Q_a/Q_c)	Peak Current Ratio (I_{pa}/I_{pc})
DMSO	-0.75	0.53	2.33	2.51	0.18	0.68	0.71
DMA	-1.6	-	2.15	-	-	-	-
DEGDME	-0.37	-	1.67	-	-	-	-
MeCN	-0.48	-	2.31	-	-	-	-

Table S5 Corresponding Solvent Raman Bands

MeCN 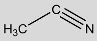		DEGDME 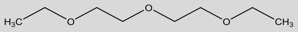		DMA 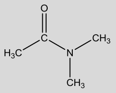		DMSO 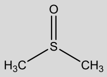	
Assignment	Band /cm^{-1}	Assignment	Band /cm^{-1}	Assignment	Band /cm^{-1}	Assignment	Band /cm^{-1}
υ_{C-C}	920	υ_{C-C}	835	δ_{O-C-N}	658	υ_{C-S} symmetric stretch	671
-	992	υ_{C-C}	860	υ_{N-C} + υ_{C-C}	865	υ_{C-S} anti-symmetric stretch	700
CH_3 rocking	1047	υ_{C-O}	1126	υN-CH_3	1096	$\upsilon_{S=O}$ symmetric stretch of dimer	1032
		υ_{C-O}	1145			$\upsilon_{S=O}$ symmetric stretch of monomer	1065

Table S6 Donor number and ^{23}Na NMR peak shift for correlated data in Figure S3

Solvent	Donor Number (kJ/mol)	^{23}Na NMR Peak shift (ppm)
Water	18	0
PC	15.1	9.5
N,N-DMA	27.8	3.91
Ammonium	59	-13.1
DMSO	29.8	0.46
Ethanol	17.1	6.7
DEGDME	18	7.2

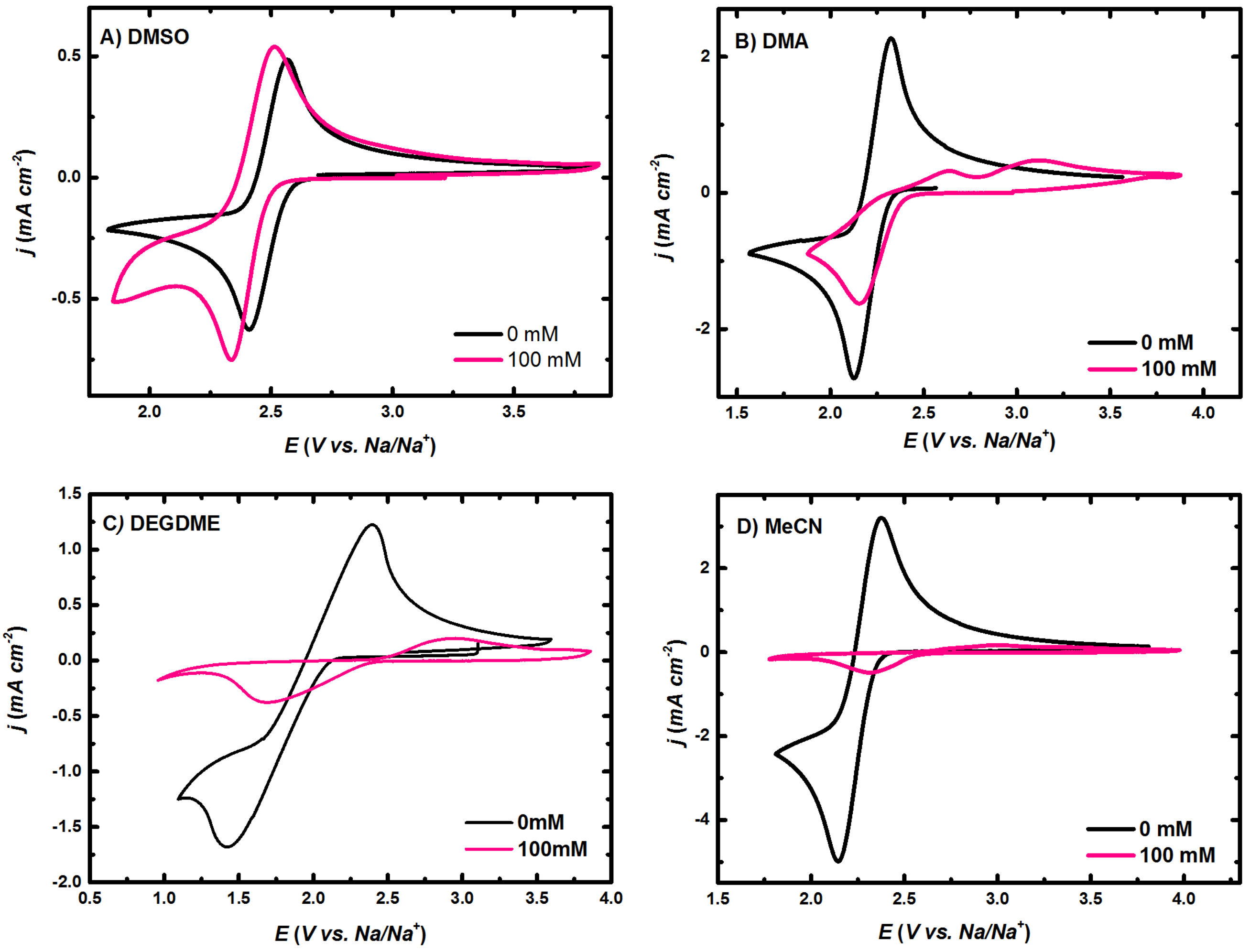

Figure S1. Voltammetric study of OER/ORR in oxygen enriched 0.1M TEA OTf &0.1M NaOTf in A) DMSO, B)DMA, C)DEGDME, D) MeCN GC, 100 mV s^{-1}, 23°C

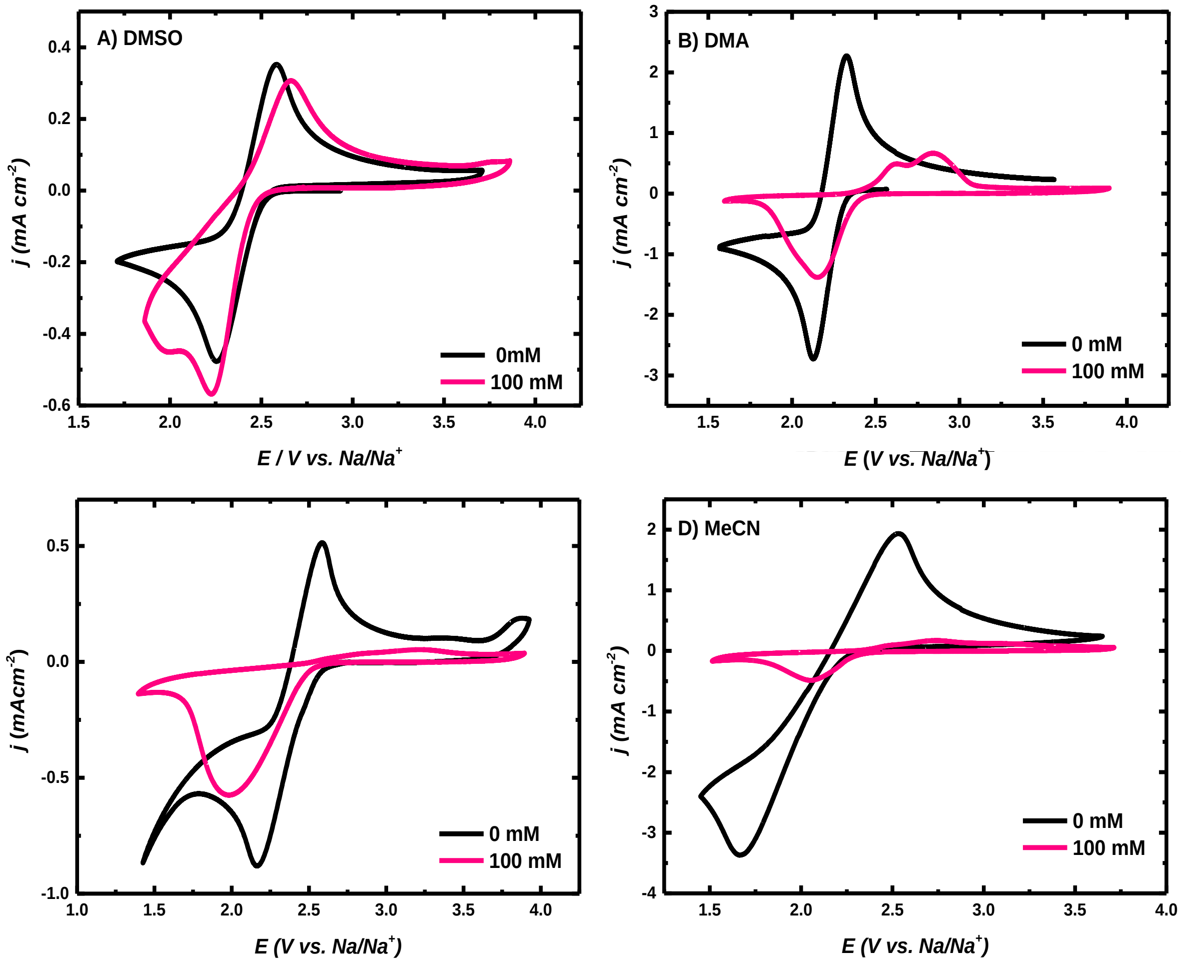

Figure S2. Voltammetric study of OER/ORR in oxygen enriched 0.1M TEA OTf &0.1M NaOTf in A) DMSO , B) DMA, C) DEGDME, D) MeCN Au, 100 mV s^{-1}, 23°C

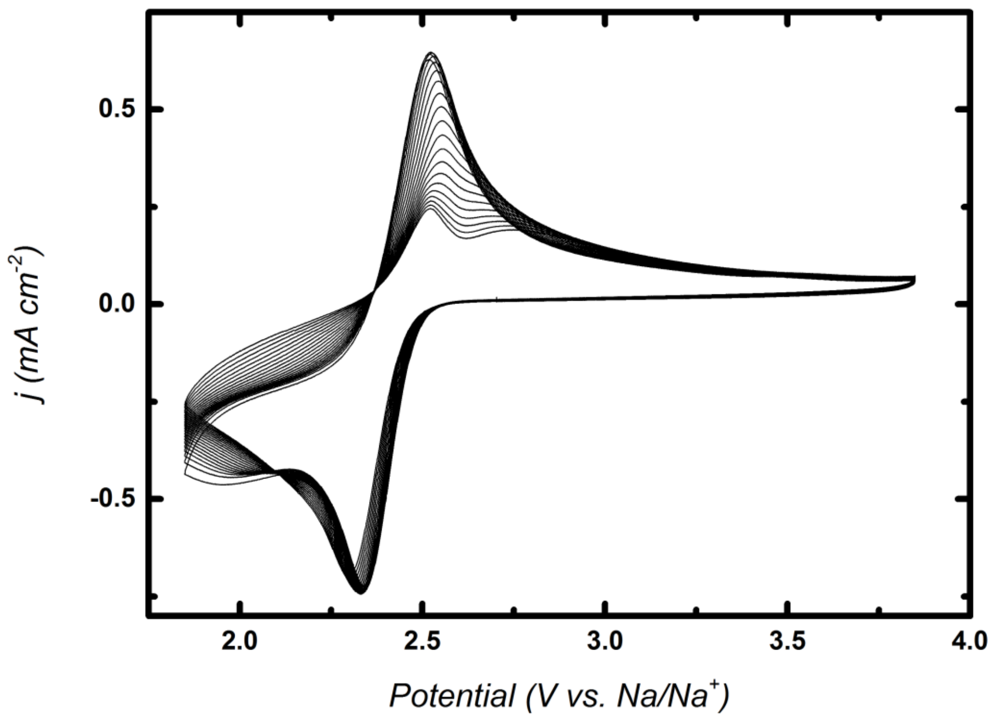

Figure S3. Voltammetric cycling (cycle 1to cycle 20 with decreasing oxidative current) of OER/ORR in oxygen enriched 0.1M Na OTf DMSO on Au, 100 mV s^{-1}, 23°C

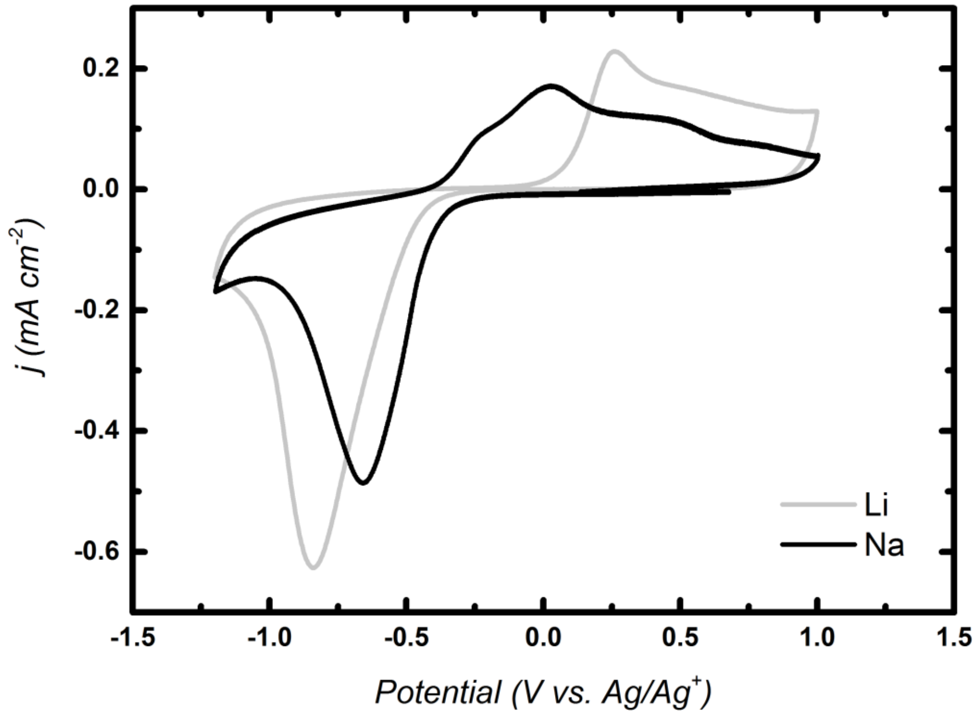

Figure S4. Voltammetric comparison of OER/ORR in oxygen enriched 0.1M NaOTf and LiOTf in MeCN on Au, 100 mV s^{-1}, 23°C

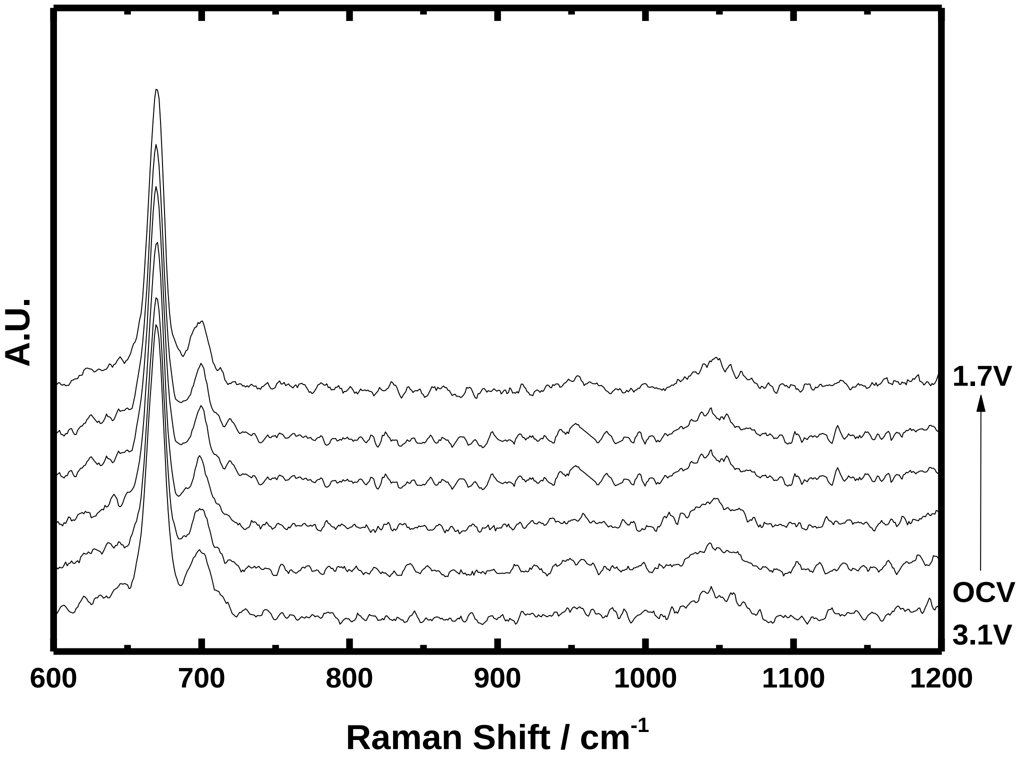

Figure S5. *In situ* SERS of Argon enriched 0.1 M NaOTf in DMSO

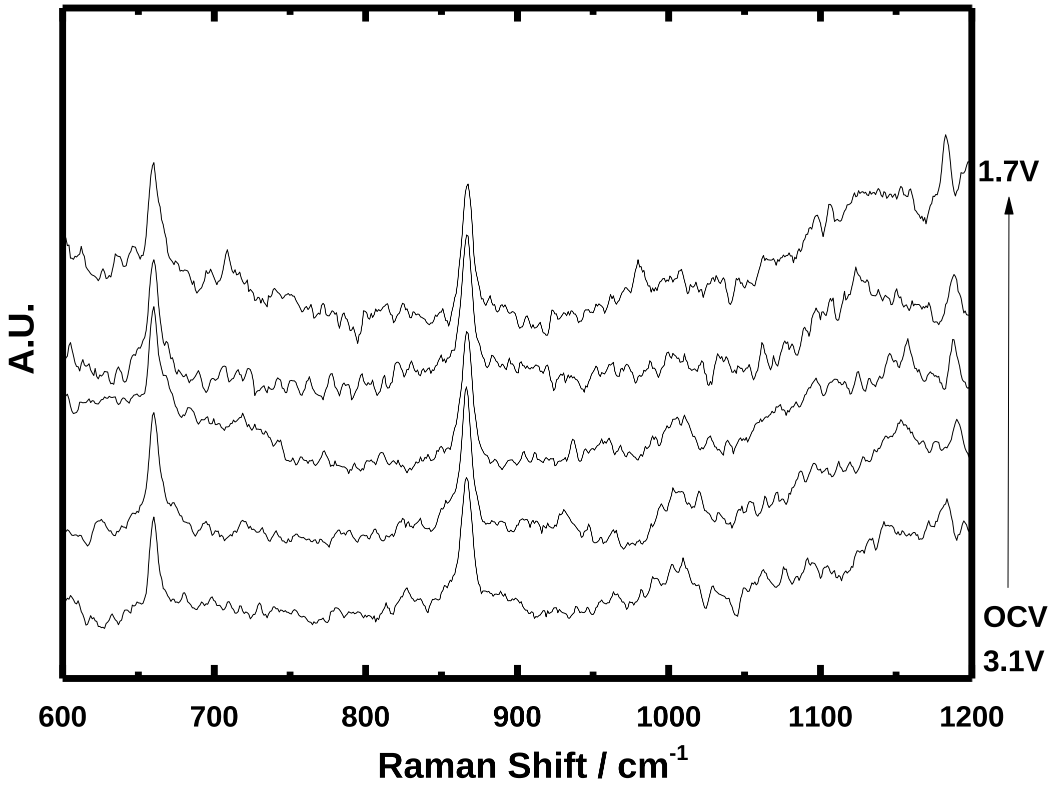

Figure S6. *In situ* SERS of argon enriched 0.1 M NaOTf in DMA

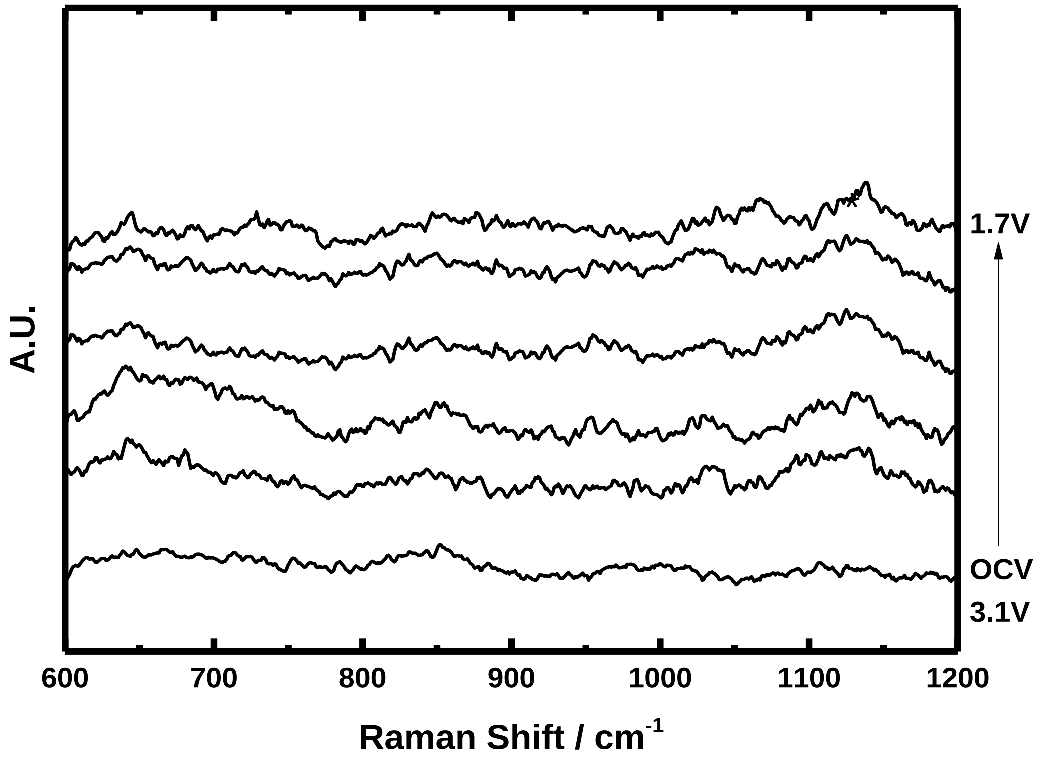

Figure S7. *In situ* SERS of argon enriched 0.1 M NaOTf in DEGDME

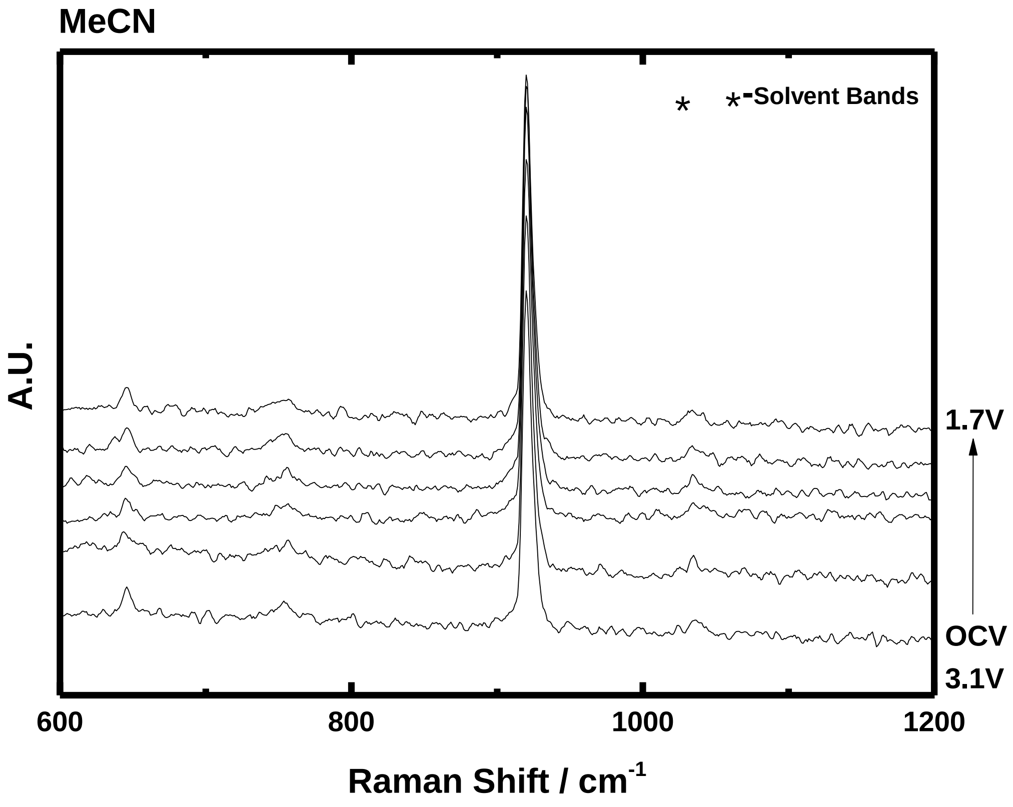

Figure S8. *In situ* SERS of Argon enriched 0.1 M NaOTf in MeCN

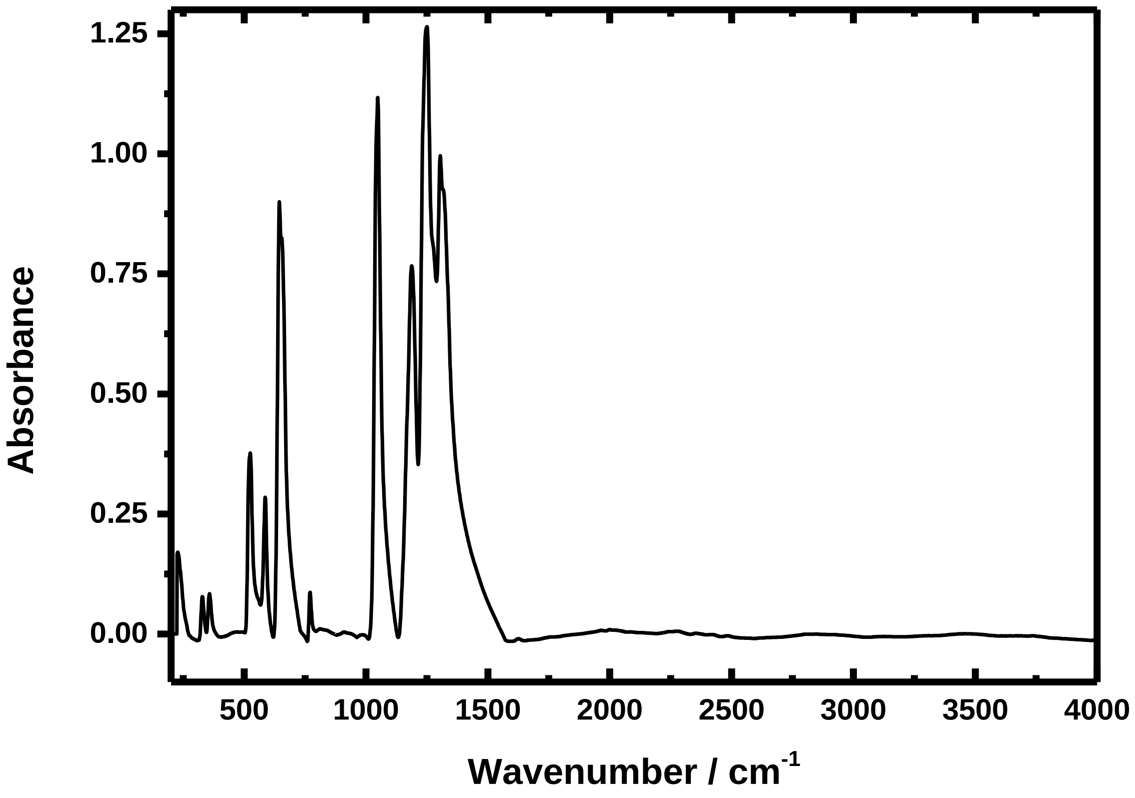

Figure S9. Infrared spectrum of NaOTf Powder (Aldrich)

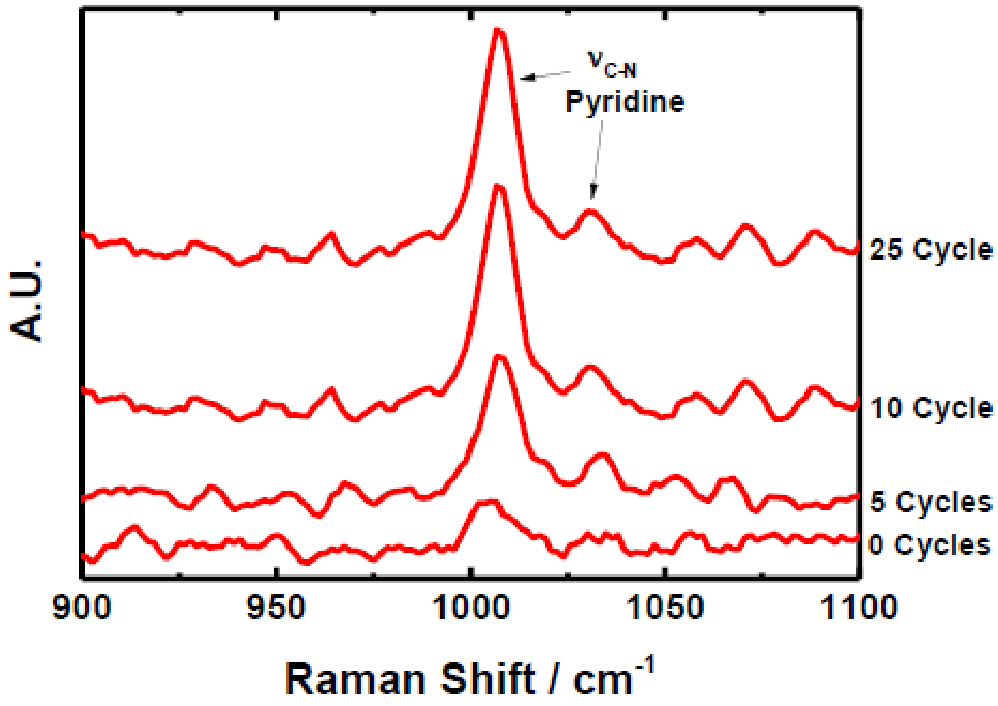

Figure S10. Oxidation reduction cycle procedure showing increasing intensity of pyridine at a Au interface

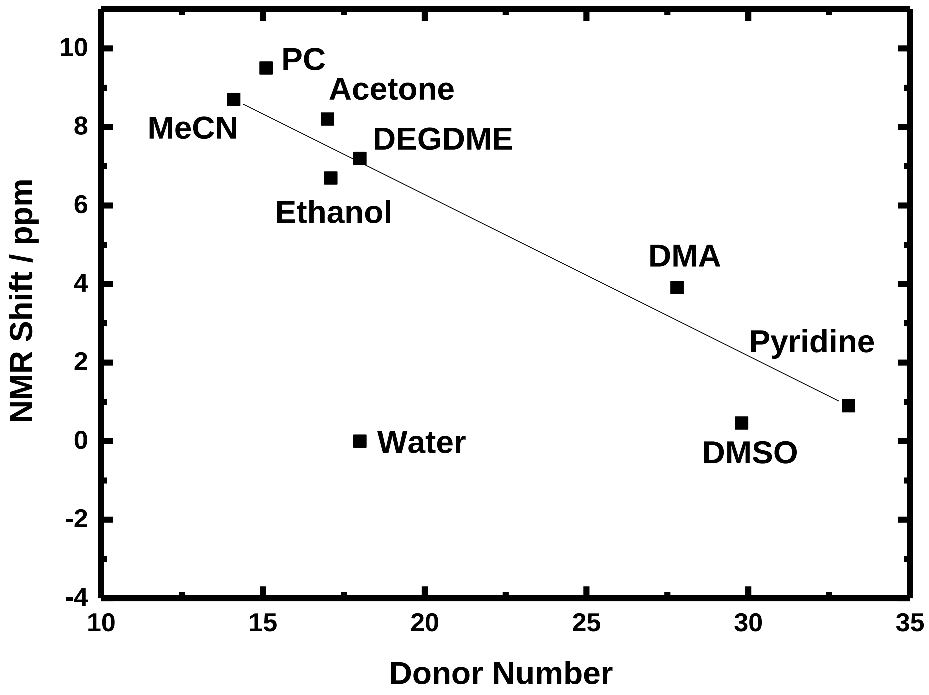

Figure S11. ^{23}Na NMR correlated to Gutmann donor number for various non-aqueous solvents and water in 0.2 M $NaClO_4$ internally referenced against 2 M NaCl

References

1. I. M. Aldous and L. J. Hardwick, *J. Phys. Chem. Letts.*, 2014, **5**, 3924-3930.
2. A. G. L. Lutz, D. Alves Dalla Corte, E. Azaceta, L. Johnson, A. Descamps-Mandine, R. Tena-Zaera, P.Bruce and J. M. Tarascon, presented in part at the U.K. - Korea Symposium on Sodium and Lithium Batteries, London, 2016.
3. L. Johnson, C. Li, Z. Liu, Y. Chen, S. A. Freunberger, P. C. Ashok, B. B. Praveen, K. Dholakia, J.-M. Tarascon and P. G. Bruce, *Nat. Chem.*, 2014, **6**, 1091-1099.
